# A Robust 6-lncRNA Prognostic Signature for Predicting the Prognosis of Patients With Colorectal Cancer Metastasis

**DOI:** 10.3389/fmed.2020.00056

**Published:** 2020-03-06

**Authors:** Shuyuan Li, Shuo Chen, Boxue Wang, Lin Zhang, Yinan Su, Xipeng Zhang

**Affiliations:** Department of Colorectal Surgery, Tianjin Union Medical Center, Tianjin, China

**Keywords:** colorectal cancer, metastasis, prognosis, long non-coding RNAs, prognostic signature

## Abstract

**Objective:** Our study aimed to construct a robust long non-coding RNA (lncRNA) prognostic signature for colorectal cancer (CRC) metastasis.

**Methods:** Differentially expressed lncRNAs were identified between metastatic CRC and non-metastatic CRC samples from The Cancer Genome Atlas Database (TCGA) using the edgeR package. The differentially expressed lncRNAs with prognosis of patients with CRC metastasis were identified by univariate Cox regression analysis, followed by a stepwise multivariate Cox regression model. The survminer package in R was used to identify the optimal cutoff point for high-risk and low-risk groups. The receiver operating characteristic (ROC) curves were plotted to assess this signature. To explore potential signaling pathways associated with these lncRNAs, Gene Set Enrichment Analysis (GSEA) was performed.

**Results:** A 6-lncRNA signature was built based on the lncRNA expression profile for CRC metastasis. The optimal cutoff value was used to classify high-risk and low-risk groups using the survminer package. The high-risk groups could have poorer survival time than the low-risk groups. ROC curve result indicated that this lncRNA signature had high sensitivity and accuracy. GSEA analysis results showed that the six lncRNAs were significantly enriched in several CRC metastasis-related signaling pathways such as “cell cycle,” “DNA replication,” “mismatch repair,” “oxidative phosphorylation,” “regulation of autophagy,” and “insulin signaling pathway.”

**Conclusion:** Our study constructed a 6-lncRNA model for predicting the survival outcomes of patients with CRC metastasis, which could become potential prognostic biomarkers, and therapeutic targets for CRC metastasis.

## Introduction

Colorectal cancer (CRC) is one of the most common malignant tumors of the digestive system, which is the leading cause of cancer-related deaths worldwide (1, 2). Because of the adequate blood supply to CRC tissues, it is easy to metastasize and invade, especially for patients at an advanced stage. Even after liver metastases resection, more than 50% of patients cannot be cured due to recurrence and distant metastases (3, 4). Tumor metastasis is a multistep cellular biology process (5). A key event in this process is the accumulation of multiple genetic and epigenetic changes (6). Despite significant advances in the diagnosis and treatment of CRC, metastasis remains the leading cause of CRC-related deaths (7). Currently, conventional treatments for metastatic CRC include resection, radiation therapy, and chemotherapy. However, these treatments have limitations such as insufficient resection and local recurrence. Radiation therapy and chemotherapy lack of tumor specificity and concomitant cytotoxic effects. Furthermore, it is difficult to treat recurrent CRC cells by conventional chemotherapy or radiation therapy. Therefore, it is important to identify genes associated with CRC metastasis.

Long non-coding RNA (lncRNA) is a non-coding RNA of >200 nucleotides in length, accounting for more than 80% of the entire genome transcript (8, 9). Increasing evidence suggests that aberrantly expressed lncRNAs contribute to various human cancers including CRC. Aberrantly expressed lncRNAs are involved in various biological processes, such as cell proliferation, cell differentiation, cell invasion, and so on (10, 11). Some abnormally expressed lncRNAs have been found to be involved in the processes of CRC metastasis (12, 13). For example, lncRNA UICLM promotes CRC liver cancer metastasis via sponging miRNA-215 (14). LncRNA AB073614 promotes CRC metastasis through the PI3K/AKT signaling pathway.

To improve the prognosis of CRC patients, several bioinformatics methods have been used to identify lncRNAs associated with CRC prognosis and to build lncRNA models. For example, Fan et al. (15) discovered a 6-lncRNA signature that can predict the prognosis of CRC patients. Wang et al. (16) constructed a 15-lncRNA prognostic model of CRC. A recent study reported an 8-lncRNA signature including PEG3-AS1, LOC100505715, MINCR, DBH-AS1, LINC00664, FAM224A, LOC642852, and LINC00662 using a random survival forest method (17). However, to our knowledge, there is no lncRNA prognostic signature associated with CRC metastasis.

Studies have shown that the performance of a single biomarker in predicting patients' survival across data sets is unstable, while the combination of biomarkers improves performance (18). In our study, we proposed a robust 6-lncRNA signature, which can accurately predict the prognosis of patients with CRC metastasis.

## Materials and Methods

### Data Acquisition and Preprocessing

The level 3 RNA-seq data of CRC including colon adenocarcinoma (COAD; 519 cases) and rectal cancer (READ; 176 cases) and matched clinical information were obtained from the TCGA database (https://cancergenome.nih.gov/). The RNA-seq data were generated on the Illumina HiSeq 2000 RNA Sequencing platform. CRC patients without complete clinical information including gender, age, TNM stage, tumor invasion, lymph node metastasis, distant metastasis, and preoperative CEA level were excluded. Furthermore, we excluded clinical samples with survival time <30 days. Finally, 546 patients with COAD and 181 patients with READ were employed for this study.

### Identification of Differentially Expressed LncRNAs Between M0 and M1 CRC Tissues

Raw count data were preprocessed using the Empirical Analysis of Digital Gene Expression Data (edgeR) in R (version 3.16.5; http://bioconductor.org/packages/release/bioc/html/edgeR.html) (19). The log-counts per million (CPM) value was used to normalize the raw count. Moreover, the genes that were expressed in more than one-third of the samples were included for this study.

The data were divided into two groups: M0 (no distant metastasis; *n* = 494) and M1 (distant metastasis; *n* = 85) CRC groups. The differential expression analysis was performed using the edgeR package in R. The differentially expressed lncRNAs were identified as follows: FDR (adjusted *p*-value) <0.05 and |log_2_-fold change|>1.

### Identification of Differentially Expressed LncRNAs Associated With Prognosis of CRC Metastasis Patients and Risk Score Calculation

Eighty-five M1 CRC samples were used as the entire set. We randomly selected 60% (*n* = 51) from 85 samples. The random selection process was performed 1,000 times. The robust likelihood-based survival model was used to identify the optimal lncRNA set. Furthermore, the frequency of lncRNAs in the 1,000 random samplings was counted. The lncRNAs with the most occurrence frequency were identified as candidate variables for the lncRNA prognostic model. After that, the risk coefficients of these candidate variables were calculated by constructing a multivariable Cox proportional hazards model using the survival and survminer package in the entire set. The risk formula was as follows: risk score = ∑ β lncRNAi × Exp lncRNAi (*i* = 1 − *n*). β indicates the coefficient of lncRNAi, and Exp indicates the expression level of lncRNAi in patients with CRC metastasis. The optimal cutoff of risk score was calculated using the survminer package (version 0.4.3) according to the expression value, the survival time, and the survival status. The patients were divided into high-risk and low-risk groups according to the optimal cutoff of the risk score. The overall survival analysis was performed between high-risk and low-risk groups. The ROC curve was used to evaluate the prognostic performance through comparing the areas under the ROC curves (AUC).

### Survival Analyses

The risk score of each lncRNA in the model was calculated in the entire set (*n* = 85). The R package survminer was used to identify the optimal cutoff of lncRNAs based on the expression value/risk score, the survival time, and the survival status. LncRNA>optimal cutoff was considered as high expression, and < optimal cutoff as low expression. After that, the correlation between the clinical characteristics of CRC metastasis patients and each lncRNA expression was performed in a univariate Cox regression analysis.

### LncRNA–RNA Interaction Analysis

To observe the functions of these lncRNAs, target proteins of these lncRNAs were obtained from the ENCORI database (http://starbase.sysu.edu.cn/index.php). Cytoscape software was used to visualize the lncRNA–RNA interaction network. These target RNAs were annotated by the Metascape database (http://metascape.org/) (20).

### Coexpression Analysis

We further analyzed the potential coexpression relationships of prognostic lncRNAs by Pearson's correlation analysis. Then, coexpression relationships were visualized.

### Gene Set Enrichment Analysis (GSEA)

GSEA was performed by the JAVA program (http://software.broadinstitute.org/gsea/downloads.jsp) using the MSigDB C2 Canonical pathways gene set collection, which consisted of 1,320 gene sets (21). Gene sets with FDR<0.05 after performing 1,000 permutations were considered to be significantly enriched.

### Statistical Analysis

All statistical analyses were performed using R. Kaplan–Meier survival analysis was performed between high-risk and low-risk groups and evaluated using a log-rank test. *P* < 0.05 was considered statistically significant.

## Results

### Identification of Differentially Expressed LncRNAs Between Distant Metastasis (M1) and Non-metastasis (M0) CRC

**Figure 1:**
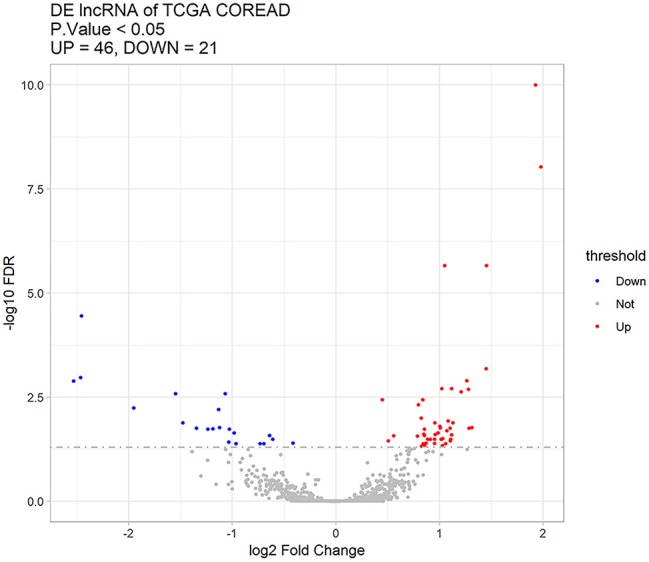
Sixty-seven differentially expressed lncRNAs between distant metastasis (M1) and non-metastasis (M0) colorectal cancer using a volcano plot. Red stands for upregulated lncRNAs, blue represents downregulated lncRNAs, and gray represents lncRNAs with no significant difference.

Sixty-seven differentially expressed lncRNAs were identified between distant metastasis (M1) and non-metastasis (M0) CRC according to adjusted *p* < 0.05, including 46 upregulated and 21 downregulated lncRNAs (Supplementary Table 1). Figure 1 shows the upregulated and downregulated lncRNAs using a volcano plot.

### Identification of Differentially Expressed LncRNAs Associated With Prognosis of Patients With CRC Metastasis

To identify differentially expressed lncRNAs associated with prognosis of patients with CRC metastasis, we performed overall survival analysis of 67 differentially expressed lncRNAs using a univariate Cox proportional hazard regression model in the entire set (*n* = 85). The lncRNAs with *p* < 0.05 were identified as differentially expressed lncRNAs associated with the prognosis of patients with CRC metastasis. Finally, a total of 18 lncRNAs were considered as candidate variables from 67 differentially expressed lncRNAs (Supplementary Table 2).

### Identification of a 6-lncRNA Prognostic Signature for CRC Metastasis

**Figure 2:**
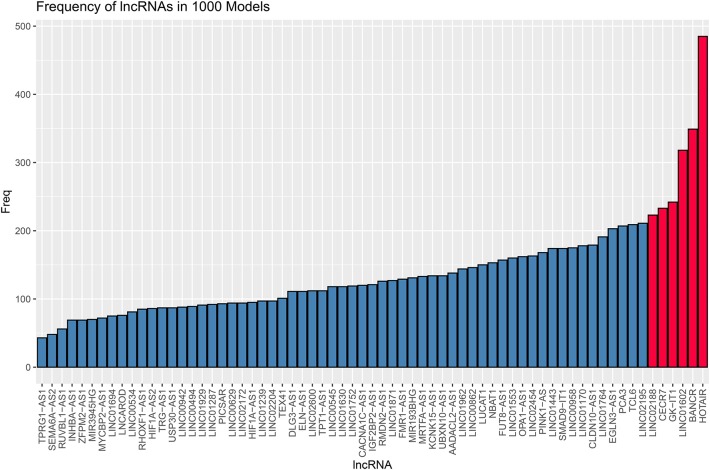
Frequency of 67 differentially expressed lncRNAs in 1,000 samplings.

**Figure 3:**
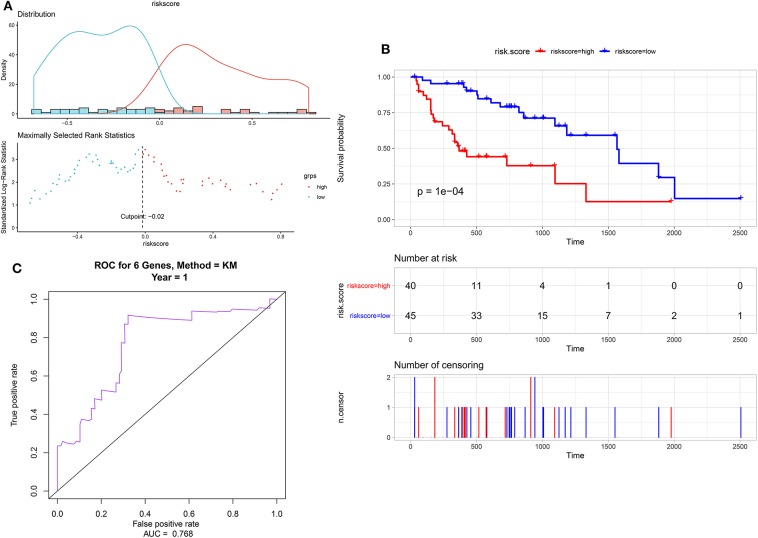
Identification of a 6-lncRNA prognostic signature for colorectal cancer metastasis. **(A)** The risk score distribution of the 6-lncRNA signature. **(B)** Kaplan–Meier survival analysis between high-risk and low-risk groups according to the optimal cutoff value. **(C)** ROC curve for predicting 1-year survival time.

Eighty-five M1 CRC samples and 18 prognostic-related differentially expressed lncRNAs were input into the rbsurv function for dimensionality reduction analysis. After random sampling 1,000 times and counting frequencies, 60% of the samples were randomly selected at a time, and the frequency statistics of related lncRNAs in the model were counted after sampling 1,000 times. As shown in Figure 2, six lncRNAs (HOTAIR, BANCR, GK-IT1, LINC01602, CECR7, and LINC02188) were retained to construct this signature. Multivariate Cox analysis of the six lncRNAs was performed, and regression coefficients were calculated in the entire set (*n* = 85). Risk score = 0.10574 ^*^ BANCR – 0.14189 ^*^ CECR7 – 0.13273 ^*^ GK-IT1 + 0.15068 ^*^ HOTAIR + 0.11772 ^*^LINC01602 + 0.01726 ^*^ LINC02188. According to the risk score, the optimal cutoff value was identified in the entire set (Figure 3A). The patients were divided into high-risk and low-risk groups according to the optimal cutoff value. We found that the patients with high risk had poorer prognosis than those with low risk (Figure 3B). As shown in Figure 3C, RUC was 0.768, suggesting that the 6-lncRNA signature had high sensitivity and accuracy.

### Cross-Validation of the LncRNA in the Prognostic Signature With Survival Outcomes

**Figure 4:**
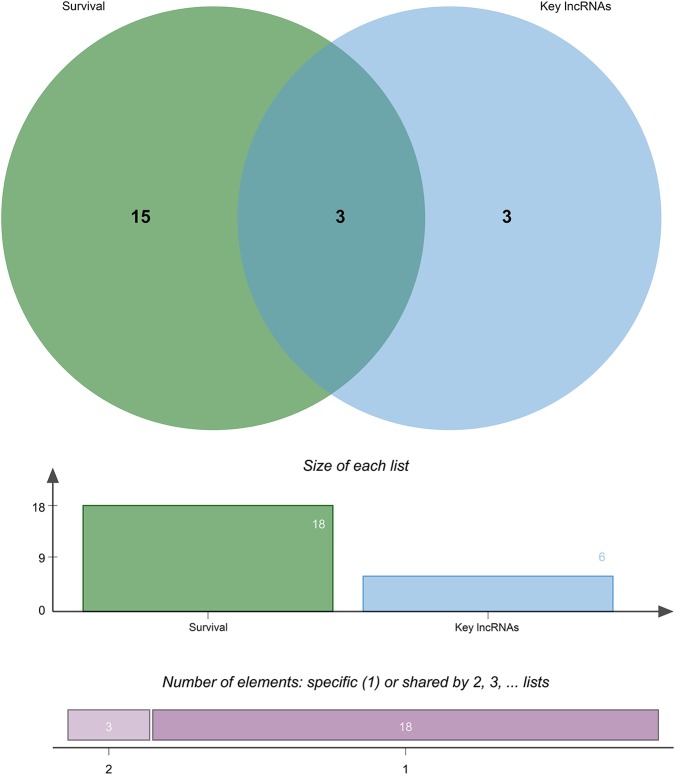
Cross-validation of the lncRNAs in the prognostic signature with survival outcomes.

**Figure 5:**
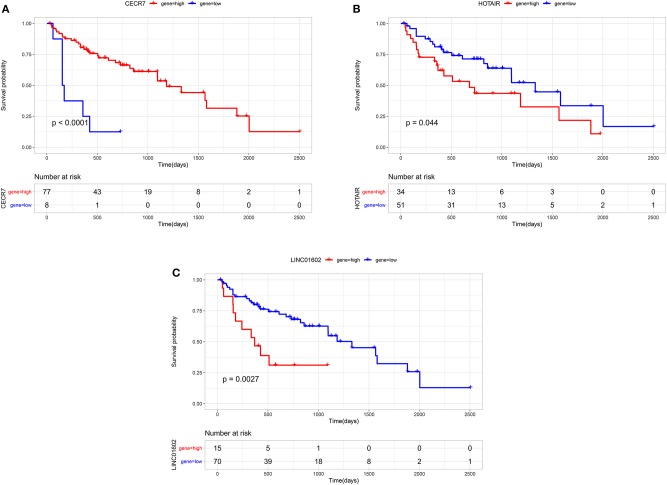
Overall survival analysis of CECR7, HOTAIR, and LINC01602 for patients with colorectal cancer metastasis. **(A)** CECR7; **(B)** HOTAIR; and **(C)** LINC01602.

Through survival analysis, out of 67 differentially expressed lncRNAs, a total of 18 lncRNAs were significantly associated with the prognosis of patients with CRC metastasis. From the 67 differentially expressed lncRNAs, we obtained a 6-lncRNA prognostic signature that could guide the prognosis of patients with CRC metastasis. Through cross-validation, we found that among the six lncRNAs in the prognostic signature, three lncRNAs including CECR7, HOTAIR, and LINC01602 were significantly associated with the prognosis of patients with CRC metastasis (Figure 4). Overall survival analysis results showed that low CECR7 expression was significantly associated with poorer prognosis than its high expression (Figure 5A; *p* < 0.0001). Moreover, highly expressed HOTAIR (Figure 5B; *p* = 0.044) and LINC01602 (Figure 5C; *p* = 0.0027) had worse prognosis for patients with CRC metastasis.

### Construction of LncRNA–RNA Interaction Network for CRC Metastasis

**Figure 6:**
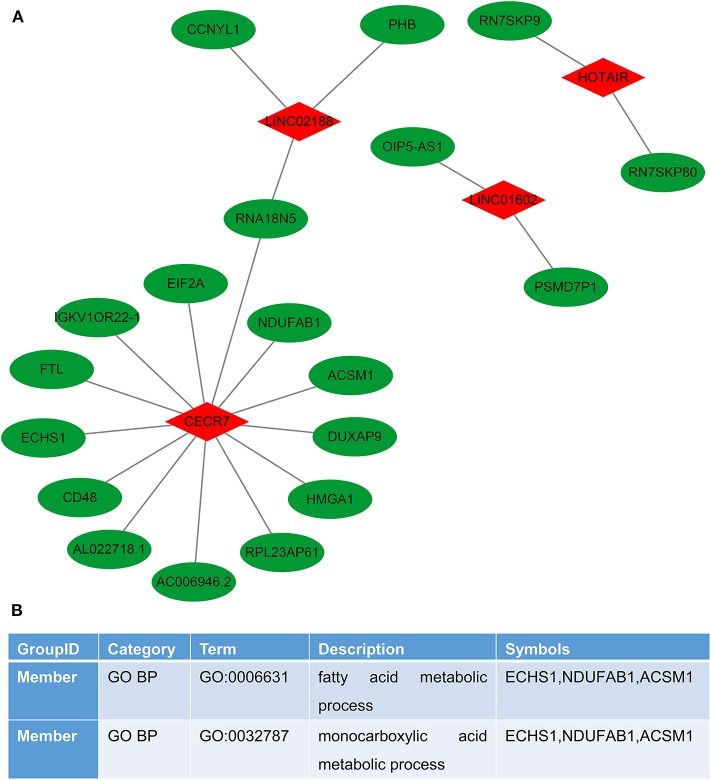
Construction of the lncRNA–RNA interaction network for colorectal cancer metastasis. **(A)** LncRNA–RNA interaction network. Red represents lncRNAs and green represents RNAs. **(B)** Function annotations of target RNAs by the Metascape database.

We performed the lncRNA–RNA interaction analysis on six characteristic lncRNAs in the prognostic signature by the ENCORI database. The lncRNA–RNA interaction network was constructed, consisting of 4 lncRNAs and 19 RNAs (Figure 6A). To explore potential functions of these lncRNAs, target RNAs were annotated by the Metascape database. The results showed that these RNAs were significantly associated with a fatty acid metabolic process and a monocarboxylic acid metabolic process (Figure 6B).

### Coexpression Analysis of the LncRNAs in the Prognostic Signature for CRC Metastasis

**Figure 7:**
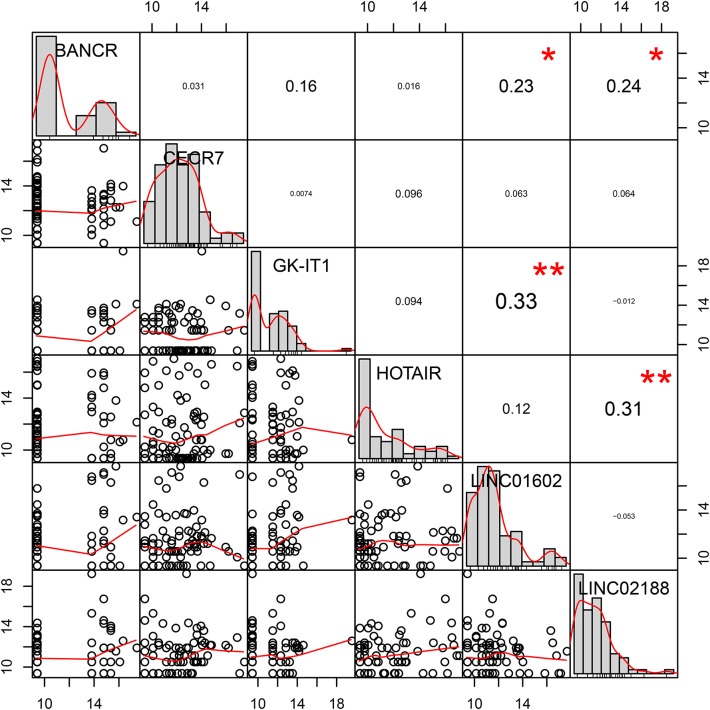
Pearson's correlation coefficients of six lncRNAs in the prognostic signature for colorectal cancer metastasis.

We further analyzed the potential co–expression relationships of the lncRNAs in the prognostic signature by Pearson's correlation analysis. The results showed that there was a positive correlation between LINC01602 and GK-IK1, LINC02188 and HOTAIR, LINC02188 and BANCR, and LINC01602 and BANCR (Figure 7).

### The Signaling Pathways Enriched by the 6-Lncrna Prognostic Signature

**Figure 8:**
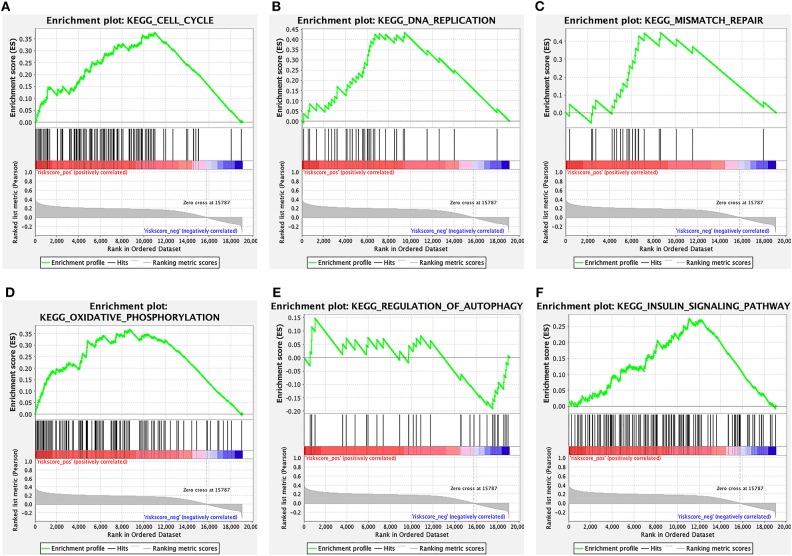
The signaling pathways enriched by the 6-lncRNA prognostic signature. **(A)** Cell cycle; **(B)** DNA replication; **(C)** mismatch repair; **(D)** oxidative phosphorylation; **(E)** regulation of autophagy; and **(F)** insulin signaling pathway.

The signaling pathways enriched by the 6-lncRNA prognostic signature were analyzed using GSEA. From the GSEA results, we found that several CRC metastasis-related signaling pathways were significantly enriched, such as “cell cycle” (Figure 8A), “DNA replication” (Figure 8B), “mismatch repair” (Figure 8C), “oxidative phosphorylation” (Figure 8D), “regulation of autophagy” (Figure 8E), and “insulin signaling pathway” (Figure 8F). The above results indicated that the 6-lncRNA signature was associated with CRC metastasis.

## Discussion

In the present study, we identified 67 differentially expressed lncRNAs for CRC metastasis based on 579 CRC patients. A model consisting of six prognostic lncRNAs that were significantly associated with CRC metastasis was established. The performance of the prognostic model was evaluated in an independent validation set and an entire set to improve its accuracy. ROC curve results indicated that the 6-lncRNA signature can predict the prognosis of patients with CRC metastasis.

As a subset of non-coding RNA, increasing evidence revealed that lncRNA is an important regulator of CRC metastasis. Our findings may provide new biomarkers for CRC metastasis. Recently, studies have reported genetic models for predicting human cancer. These studies have developed different genetic models using different methods. For example, 3-lncRNA signature (CAT104, LINC01234, and STXBP5-AS1) was conducted for predicting the prognosis of breast cancer (22). However, the patients were divided into low-risk and high-risk groups using the median risk score as the cutoff point, which could be not accurate enough. In the present study, our research used a stepwise analysis. First, metastasis-specific lncRNAs were identified in a large number of CRC patients. Sixty-seven lncRNAs were differentially expressed between metastatic CRC and non-metastatic CRC. Among them, survival analysis showed that 18 lncRNAs were associated with the prognosis of patients with CRC metastasis. Next, six lncRNAs were screened for the construction of the 6-lncRNA model by univariate and multivariate regression analysis. More importantly, the optimal cutoff was calculated to classify high and low expression groups according to the gene expression value /risk score, the survival time, and the survival state. Compared with the traditional methods such as the median value of risk score, the optimal cutoff can more accurately classify high and low expression groups (23–25). Survival analysis revealed that the risk score model could significantly distinguish the prognostic differences between high expression and low expression groups.

Among the six lncRNAs in the model, HOTAIR has been confirmed to be associated with CRC metastasis. For example, it has been reported that HOTAIR can mediate CRC tumorigenesis and liver metastasis *in vivo* (26). Furthermore, HOTAIR could promote CRC cell proliferation, migration, and invasion via sponging miR-197 (27). Consistent with a previous study, high HOTAIR expression indicates poor prognosis for patients with CRC metastasis (28). BANCR has been found to be involved in CRC migration through mediating epithelial–mesenchymal transition (29). High expression of BANCR could be in positive correlation with CRC metastasis and poor prognosis, which was consistent with our findings (30). Consistently, it has been reported that LINC01602 could predict poor prognosis of CRC patients (31). However, the mechanisms of GK-IT1, CECR7, and LINC02188 in CRC metastasis remain unclear. We found that GK-IT1, CECR7, and LINC02188 had significant correlations with the prognosis of patients with CRC metastasis. Therefore, these lncRNAs could be considered as biomarkers of CRC metastasis. GSEA analysis results showed that the six lncRNAs could be involved in CRC metastasis by several cancer-related signaling pathways, such as “cell cycle” (32); “DNA replication” (33); “mismatch repair” (34); “oxidative phosphorylation” (35); “regulation of autophagy” (36); and “insulin signaling pathway.”

In conclusion, we conducted a 6-lncRNA model for predicting CRC metastasis, including HOTAIR, BANCR, GK-IT1, LINC01602, CECR7, and LINC02188. The 6-lncRNA model could accurately predict the prognosis of patients with CRC metastasis.

## Conclusion

In our study, we constructed a 6-lncRNA signature for predicting the prognosis of patients with CRC metastasis. We found that the 6-lncRNA model could possess potential value for predicting the prognosis of patients with CRC metastasis. In addition, these lncRNAs could be involved in several pathways associated with CRC metastasis.

## Data Availability Statement

The datasets analyzed during the current study are available from the corresponding author on reasonable request.

## Author Contributions

XZ conceived and designed the study. SL, SC, and BW conducted most of the experiments and data analysis, and wrote the manuscript. LZ and YS participated in collecting data and helped to draft the manuscript. All authors reviewed and approved the manuscript.

### Conflict of Interest

The authors declare that the research was conducted in the absence of any commercial or financial relationships that could be construed as a potential conflict of interest.

## Supplementary Material

Supplementary Table 1 The 67 differentially expressed lncRNAs between distant metastasis (M1) and non-metastasis (M0) colorectal cancer.

Supplementary Table 2 The 18 differentially expressed lncRNAs associated with prognosis of colorectal cancer metastasis.

## Abbreviations

lncRNAs: long non-coding RNAs
CRC: colorectal cancer
TCGA: the cancer genome atlas
ROC: receiver operating characteristic
GSEA: gene set enrichment analysis.
